# Fischer Linear Discrimination and Quadratic Discrimination Analysis–Based Data Mining Technique for Internet of Things Framework for Healthcare

**DOI:** 10.3389/fpubh.2021.737149

**Published:** 2021-10-12

**Authors:** Mohammad Kamrul Hasan, Taher M. Ghazal, Ali Alkhalifah, Khairul Azmi Abu Bakar, Alireza Omidvar, Nazmus S. Nafi, Johnson I. Agbinya

**Affiliations:** ^1^Center for Cyber Security, Faculty of Information Science and Technology, Universiti Kebangsaan Malaysia (UKM), Bangi, Malaysia; ^2^Skyline University College, University City of Sharjah, Sharjah, United Arab Emirates; ^3^Department of Information Technology, College of Computer, Qassim University, Buraydah, Saudi Arabia; ^4^Engineering Department, Tehran Urban and Suburban Railway Co., Tehran, Iran; ^5^School of IT and Engineering (SITE), Melbourne Institute of Technology, Melbourne, VIC, Australia

**Keywords:** data mining, Fischer linear discriminant analysis, quadratic discriminant analysis (QDA), feature exaction and selection, Internet of Things—IoT, healthcare applications

## Abstract

The internet of reality or augmented reality has been considered a breakthrough and an outstanding critical mutation with an emphasis on data mining leading to dismantling of some of its assumptions among several of its stakeholders. In this work, we study the pillars of these technologies connected to web usage as the Internet of things (IoT) system's healthcare infrastructure. We used several data mining techniques to evaluate the online advertisement data set, which can be categorized as high dimensional with 1,553 attributes, and the imbalanced data set, which automatically simulates an IoT discrimination problem. The proposed methodology applies Fischer linear discrimination analysis (FLDA) and quadratic discrimination analysis (QDA) within random projection (RP) filters to compare our runtime and accuracy with support vector machine (SVM), K-nearest neighbor (KNN), and Multilayer perceptron (MLP) in IoT-based systems. Finally, the impact on number of projections was practically experimented, and the sensitivity of both FLDA and QDA with regard to precision and runtime was found to be challenging. The modeling results show not only improved accuracy, but also runtime improvements. When compared with SVM, KNN, and MLP in QDA and FLDA, runtime shortens by 20 times in our chosen data set simulated for a healthcare framework. The RP filtering in the preprocessing stage of the attribute selection, fulfilling the model's runtime, is a standpoint in the IoT industry.

**Index Terms:** Data Mining, Random Projection, Fischer Linear Discriminant Analysis, Online Advertisement Dataset, Quadratic Discriminant Analysis, Feature Selection, Internet of Things.

## Introduction

The importance of information technology in the age of communication is not hidden from anybody. In 2021, more than 35 billion things are connected, and lots of potentials can be utilized in Internet of things (IoT) healthcare applications. It is not unrealistic to conceive that in the near future, artificial intelligence can substitute the service-based evaluators and manipulate IoTs that are much more accurate, professional, and appealing instead of ones with various privacy gaps. They can be accessed through a form of robots that hijack their information, resulting in incorrect information circulation and also increasing the riskiness of information of healthcare services. The third wave Web x.0 is now evolving by enhancing the Internet capacity in which companies today use the fourth generation of the IoT for their enterprise resource planning (ERP) applications. This generation has reached cloud computing, multiplying its capabilities in the virtual world in terms of velocity, veracity, and variability in crowdsourcing projects, such as grid.to, or other crowdsourcing events in cyberspace, which has achieved a high synergy in terms of availability and responsiveness. The best learned lessons on services such as Uber—which already managed to attract millions of visitors—was the reason that motivated us to perform a classification algorithm on this data set of web usage in which many points of view, such as the ethical, professional, civilian's rights, and media laws, are likewise healthcare data sets and should be taken into account.

Online advertisements in widgets, banners, and plugins are always being updated by the service providers. Their components are now coded by XML^1^ or AJAX^2^ compilers acting on the service side, leading to optimization of the server idle time. These web services are often programmed in PHP or other frameworks, such as Perl or CSS. But the design, maintenance, and updating of such services are necessarily done as separately managed projects, in which the result is aggregated in the service according to the perception of end users and other stakeholders. For instance, the Bitcoin exchange intermediaries generally compete in a win–win or offensive strategy for advertising luxury brands, soccer competitions through reputable sponsoring channels, and specialty stores and branches on free trade zones to enter the market decentral delivering the service at any location. Here, Bitcoin miners and exchangers are thought to be among the most critical stakeholders in the affluent class. Their emotional behavior is accompanied by their high expected reliability and tangibility of the service at very high prices. During the operation of Digital Wallet, many stakeholders decide to research on the queuing systems (1), maintain their privacy on attacker behavior, or change their service's ERP system by taking advantage of a new big data system on security concerns (2, 3).

We have selected this standpoint for the selection of our data set. Therefore, in this research, we presume that the web usage features are among the most important topics in the scope of the IoT and would be studied especially for healthcare frameworks with machine learning.

In this research, the ultimate goal is to study the dynamics of the IoT-intensive systems for data mining resources, such as runtime and responsiveness, and also in the essential part of analysis regarding the transparency of structure and reliability measured by accuracy. Therefore, analysis of modeling focuses on the accuracy of Internet advertisement recognition and relies on the runtime on the unstructured high-volume data as an example of IoT systems. In this paper, we first review the literature in the field of web usage and data mining. Then, the ethical and professional aspects are discussed to link the framework to practice. Finally, the results of the modeling are represented and compared with recent works in this area. The work outlook is discussed in the conclusion, in which the proposed methodology challenges can be aggregated to IoT evaluation frameworks such as service empathy, user-friendliness, responsibility, trust, and tangibility. This leads especially to achieve the solution for startups in the IoT industry in the legal, social, and even more professional sectors, such as health.

## Ethical and Professional Issues in IoT

Nowadays, online user experience has emerged among healthcare and IoT systems, demonstrating a set of secure feature locations to other applications (4). These links could be another application or a social network that is a place to discuss and critique the product or a link to a vendor partner or a public health information panel.

How do different brands in the advertising industry measure their effectiveness in online advertising and their limitations in this way, and how do they overcome it? Principally, the answer to this question depends on customer feedback. This is because customer feedback results from the customer interaction with the service through an omnichannel. Google is using web analytics widgets to display complete information about web historiography. The same widgets for details about corporate websites include a list of links to a specific brand's website announcing some related keywords (5).

In an online advertisement, another thing that brand owners consider is the emotional aspects of the customer. The customer's memories, feelings, and mood when he or she received the product or service make him or her buy again. A dynamic co-creation framework is recently developed to measure trust as a good advertising motive for adding value (6). The trust can be viewed from two dimensions, namely, the dimensions of affectional and behavioral trust. Affections are related to emotions and the atmosphere in which the service is experienced, and consumer behavior is subjective and is specific to the personality. Affectional trust has opened a new way to effectively connect customer feeling to the space in which advertisement is taking place. This can effectively be implemented in the IoT systems when the users trust the quality of service in that framework. Establishing a relationship with the customer can be inspired by new ideas and can be considered an advantage. This connection plays a significant role, especially among startups that do not specialize in the IoT industry. By any innovation, the campaigns and technical events promote ideas that may be forgotten. This is when startup dictionaries may come across terms such as internal financing, mass outsourcing, and collaboration.

**Figure 1:**
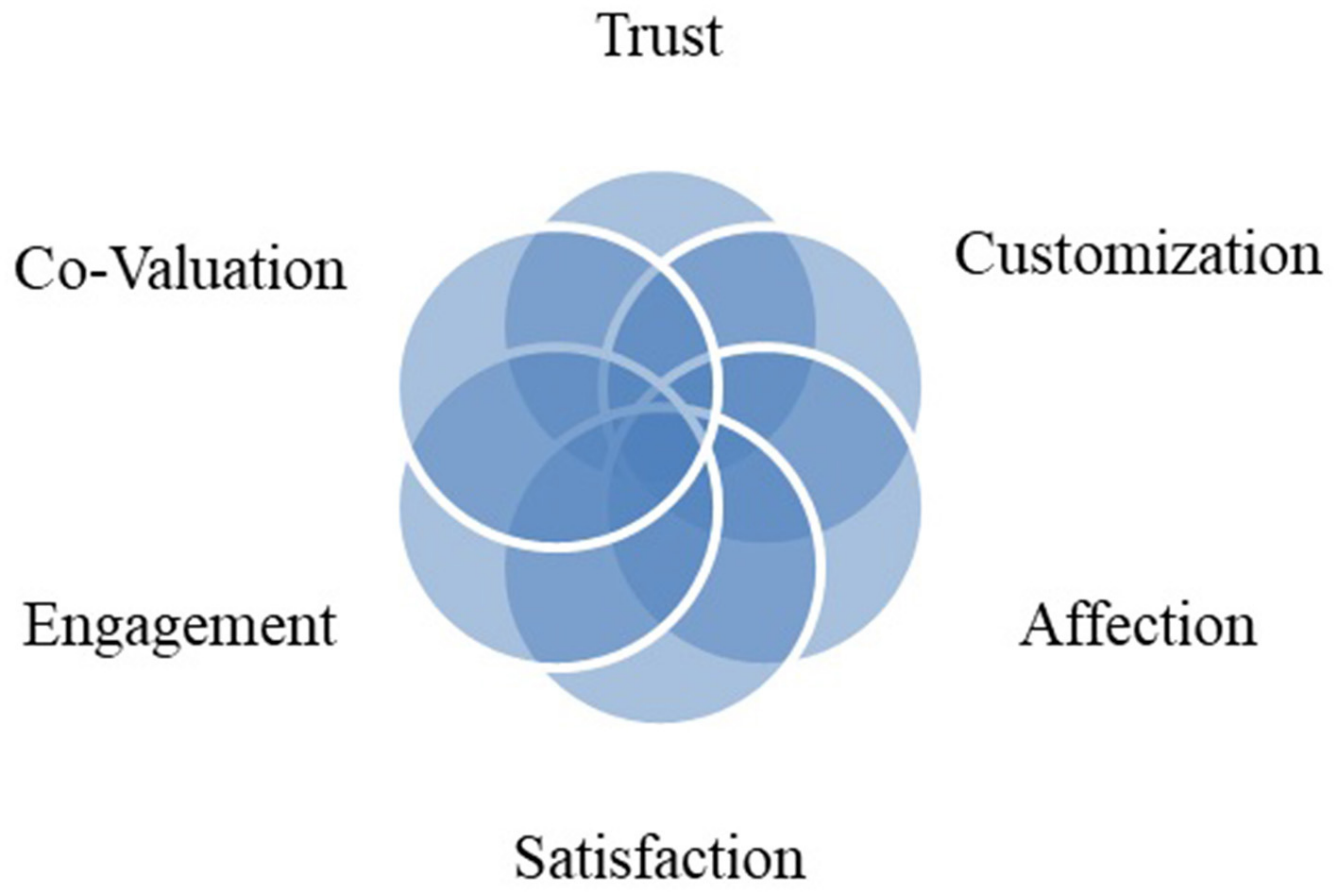
Relationship of e-shared value, trust, and affection (7).

**Figure 2:**
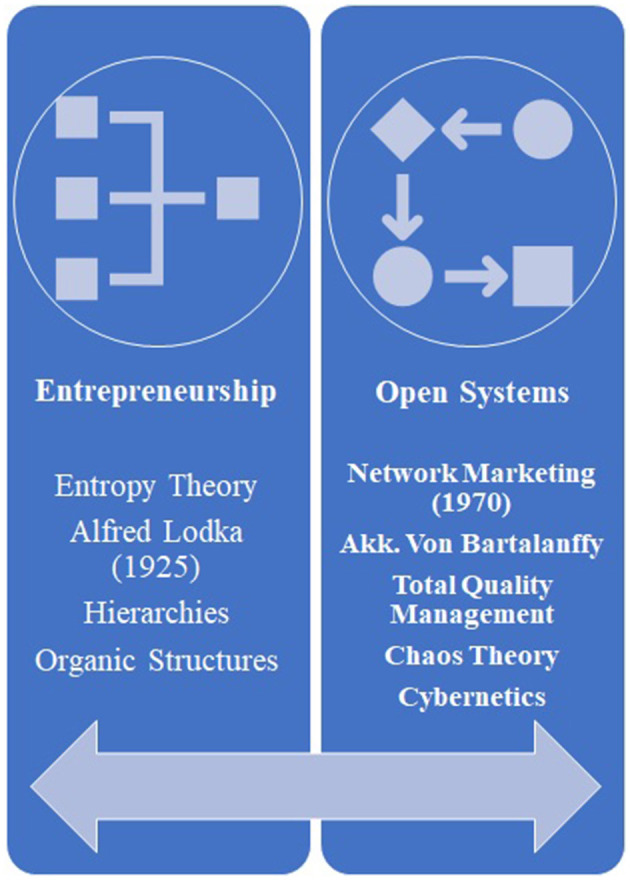
Historical development of network marketing as the basis for the online advertisement.

There is the same feeling from the brand owners' point of view, and he will take the necessary profit from the competitive advantage created by affective communication to the satisfied consumer. Brand loyalty is derived from a sense of satisfaction that is the result of creating shared value. The model in Figure 1 illustrates this relationship schematically (7). Another dimension is the interaction between brands and the customer, which causes the customer to engage with the product's contents, the function of the product in terms of professional and technical specification, and even its working model for the first time on the web. Therefore, website communicators ought to complete this connection with a proper website's proper personalization, such as flexible design. Advertisement appealing capabilities are then crucial to control this interaction. In other words, customer engagement is a set of subprocesses that use customer experience in online brand communities and value creation. It is worth noting that history helps examine the background and origins of how network marketing is created (Figure 2). In 1925, the theory of open systems was criticized by Alfred Luca in the study of organic systems by many researchers. Fifteen years later, the idea of an open system was improved by Ludwig Von Bartalanffy, who introduced it in management, which is widely used in entrepreneurial systems today (8). This is how Peter Singe, as the father of management science, defines learning systems in his book *The Fifth Discipline*. Advances in management science, and especially knowledge management, show that all processes can be defined as knowledge-based processes.

“The process of leveraging or customizing existing knowledge to meet the needs of users who have this personal need and seeking to create and innovate new knowledge” (9)—today, the blue ocean strategy is rooted in this concept after the emergence of cyber business only two decades after the management of comprehensive systems and chaos theory was entered into the post-modernism management theory. Researchers in cyberspace have recently observed that the collaboration space or distance working leads to even higher productivity as an emerging phenomenon called fast advertisement. When a high amount of cost is saved and the promotions are effective, they tend to use Internet cryptography to collect essential information from their consumers by the specified level of difficulty. Using online advertising technology should help this industry know users' opinions and their partners. It is worth noting that even non-consumers of an IoT encourage this competition, which creates a form of conflict between different parties and increases their attractiveness. For example, according to a published press “Binance” Exchange, well-known Bitcoins exchange mediaries experienced a security breach and were hacked on May 7, 2020. Hackers use various methods, such as phishing, to obtain large customer information such as 2FA codes and API keys, among other information (10). The hackers intend to change the price of Bitcoin, and even they were able to withdraw 7,000 bitcoins (BTC) worth more than $40 million at that time, and they controlled only 2% of the exchange resources. These frauds turn into severe conflicts and problems in the long run.

This question may also be considered in online advertising given that some companies that have branded in the field of online have also entered the field of off-line. For example, Bitcoin miners from popular website Lootbits have reached off-line activity and now operate as a gift promoter machine for soccer lotteries. Conversely, as with Paxful, there are companies that have gone online from the field of exchange business, and in the meantime, some prefer to choose a new name for their new brand, but in these cases, opponents of the existing brand believe that consumers like to register a new brand online. As a result, it subconsciously induces another brand field, shows the company's expertise, and strengthens the new name base. On the other hand, it automatically encourages the brand's stakeholders or the opponents of the new name for the brand to believe the cost of branding and the new name for the brand.

Coworking and B2B meeting groups have made significant progress in establishing virtual business offices, social networks, and telecommuting and collaboration. The argument here among startup managers is that only 1 in 10 companies succeed by developing a suitable business strategy. We should use a planner. After designing it, we can focus on a small part of the market share by differentiating or breaking the cost.

## Literature Review

In IoT with a technological enabler, such as big data, reliability and trust have evolved a new definition. Traditionally, IT governance in the deepest layer was hierarchical. It caused lots of information silos and other inefficiency gaps. From a bottom-up point of view, citizen- and community-driven innovations are mostly needed by some stimulations and promotions. The emerging initiatives such as “Open City Dialogues” along with the government's requirements on transparency create a paradox that can best be handled by decision and policy makers (11). We believe that IoT end service users are mostly concerned about tangibility, accountability, and empathy of the service they receive. In the near future, the citizens will be experimenting with socio-regenerative developments and with emerged society rituals. Therefore, it is not surprising that incubators and sponsors of such crowd-sourced services contribute in this era, especially in the field of IoT (12).

Some of the more recent advances are using task allocation and privacy preservation algorithms. A heuristic called data aggregation error minimization (DREAM) in privacy-preserving crowdsensing uses Liyapunov stochastic programming with the random behavior of arriving sensing tasks (13, 14). It minimizes the data aggregation error of sensed tasks while preserving the privacy for a user engaging the sensing tasks.

The scientific trend of IoT has not yet finished. It covers a wide range of topics, such as neural networks, meta-heuristics' algorithms, random walks, systems dynamics, or probability and mechanical statistics. These frameworks and modeling have been recently cited through various methods and criteria in web usage, too (15–20).

For scientific research in data mining, the Yao et al. study can be mentioned in which the probability theory of websites was intended (18). In this research, it is stated that the pure usage of a website is equal to the sum of the rankings of the link pages of that website. This notation is solved by the method of Markov chain by the random walking dynamic programming algorithm. In this research, according to the experimentation of several websites with.gov extensions, the integrated methods of complexity coefficient are compared with each other. The Euclidean distances were chosen as the measurement method, and the aggregation of the ranking make it possible to accurately estimate the out-of-sample error.

In another study search engine, keywords were used for the products that were sold an e-commerce firmware (21). These rankings were then evaluated by a structural algorithm called a web dictionary, in which the frequency of that word usage was measured. For obtaining the website evaluation the retention time of users, the minimum and maximum length of the keyword, the amount of feedback, and semantics of the website were connected via a neural network applying five input layers.

In addition to using the above semantic algorithms, graph theory is also accompanied by extracting the number of webpages in the log as the nodes and the external links as the facet forming a graph. The evaluation of the website traffic activities was determined by a eugenic value vector using the chain relations of the Markov model evaluation of the website traffic activities determined by a eugenic value vector using the Markov model's chain relations. It was observed that the resulting model was convex. By calculating the eugenic factor of the site's establishment date, taking website updates and the site ranking relationships into account, the problem was formulated, and their time weighting in terms of added value was found.

Identifying the criteria for the success of websites through a questionnaire survey that positively increases the service level is mentioned elsewhere (22–24). Criteria, such as overall website performance, reliability, information content, and source credibility are examined separately, and the effects of other unseen characteristics were experimented. To answer the research question, structural modeling is proposed. Finally, 13 components of this study are reduced to four elements by principal component analysis (PCA). Ultimately, high-ranking websites are determined out of the 80 e-commerce websites belonging to Persian Gulf countries. In a controlled manner, these criteria are selected and finally ranked by selected individuals from selected countries with high Internet penetration rates in their countries, such as Iran, UAE, and Qatar. This data is then used to provide a theoretical framework for ranking e-commerce websites. The four elements mentioned are attractiveness, competition, engagement and retainment, indicating the amount of marketing aspect vs. the website's attractiveness at first sight.

A challenging problem in the integrated data set and the high data volume that failed in the works, as mentioned earlier, is fraud sensitivity. The data sets meet the highest standard security level by applying the most elevated standard security level with state-of-the-art machine learning to detect changes according to the algorithm's patterns. There are two methods for fraud detection: an on-the-fly schema discovery of data patterns on the client side and a solution on the data digest between the servers. Data set integration as the promising characteristics of IoT systems has enabled high security, especially in world disaster times such as the trends in virus prepandemic peak. It seems that dynamic effects are still unknown, but an agentless system, fortunately, focuses on architecture planning with monitoring an agentless system to overcome security frauds. Anyway, in intrusion cases, it is practical to perform security checks with ICMP and Ping. Both methods mentioned suffer from the low level of transparency resulting in risking usage, sharing, and processing of data sets by third parties (26, 27).

From the security point of view, this research can also explain how to detect anomalies in networks for a learning algorithm that can handle the training and testing of the data sets by an ordinary classifier (25). In this research, a binomial classification model was developed that can detect if LAN activity is in the benign or attacking category based on different attributes. The data set that is publicly available, the KDDCUP data set (28), could be used as the learning data set for testing machine learning in IoT systems. Application of different models and methods can be advantageously used for our research data set to compare the result with the advertisement sets (29). Other research models, such as the one reported in the literature, can also be helpful for the purposes of comparison accuracies (30). This data set is also discussed in the open-source data repositories by different tools and methods, and the F-score, a measure for the practical calculation of the accuracy with the consideration recall of the results, is reported to be between 0.94 and 0.96. We believe that, by selecting the appropriate preprocessing and filtering and cleaning the raw data set, it is possible to outperform this accuracy. It is also beneficial to hybridize the different algorithms to operationalize the same results by the least calculations and computational resources (31).

A more recent data set that was accessed from the Kaggle platform in our research is based on categorization of the attacks according to their types (32). This system's advantage relative to the binomial classes is that few overfitting risks are in the final results. The question is still unanswered as to how deep one can define each category interface to ensure the reliability of the result. Because no retrieving log file activities should be perceived as an attack, in this case, we assume that there is a difference between in- and within-group variance by the *p*-value estimates.

The other approach considered a successful one is to collect customers' intentions by mining the web server's logs to find the most linking visiting websites (17). The authors propose the use of a fuzzy web advertisement selector. Their methodology is divided into three steps: target customer clustering, fuzzy membership, and Web ad matching. For customer clustering, the authors use the neural network technique to discover backend knowledge of the web server's log files and cluster customers into different categories based on their interest. By using fuzzy rules in fuzzy inference, the authors determine the customers' categorized viewing patterns. Then, the fuzzy inference feeds the web ad selector with these patterns and enables web ad matching to suggest the corresponding advertisement to the target customer. The authors have tested their method by implementing a fuzzy web ad selector in a newspaper website. To verify the effectiveness of the fuzzy web ad selector; the authors compare click-through rate (CTR) values before and after using their method. Thus, the authors divided the newspaper web servers into two groups. One is using a web ad selector and is called the “treatment group,” and the other is not using the web ad selector and is called the “control group.” After running the method in newspaper websites for 2 months; there was an increase in CTR values for most sections. Businesses recognized the importance of identifying customers' needs and interests in order to provide them with right product and services. Thus, businesses are using a recommender system built to advertise products based on customer preferences. These systems are using transaction recorders, web logs, and cookies to learn customers' interests. Recommender systems are using two approaches: a content-based and a collaborative approach. A content-based approach is dependent on customer feedback. However, the collaborative approach depends on examining users' relevance and selecting a recommended partner for the target user. Thus, the collaborative approach advertises products to target users who appear in the recommendation partner only. These two approaches have some limitations and problems, such as having no rating or feedback for new products and the rating is based on the customer point of view and style and cannot be a standard for all users.

To overcome these limitations, the authors propose a new data mining framework that is based on social networks (33). Social networks consider a rich data set that contains a lot of information can be used for advertising systems. The authors use data sets containing customer connections and those that involve customer transaction recorders. The authors use the first data set to identify customers' subgroups (each subgroup has a group of customers who know each other as friends, relatives, coworkers, etc.). The second data set is used to predict products' liking rate according to customers' past transactions. To use both sets, the authors develop a cohesion algorithm that is used to measure the number of customers sharing common interests for each subgroup based on their past transactions. This framework can target new customers by identifying to which subgroup they belong and verifying the liking rate for that subgroup. The authors have tested their proposed framework by targeting book advertising for their university library. The authors built their own social network by using email logs for their university facilities and circulation data for facilities from the library. The authors compared their approach (group-based) with other approaches: the single-based approach (assuming each user as a group); neighbor-based approach (grouping each user and his direct neighbor); and random, which randomly chose users. This study proves that their proposed framework outperforms the other approaches.

Personalizing advertising becomes a challenge for many electronic commerce companies because of the increasing number of Internet users. In addition, companies are looking to personalize advertising to reach the maximum number of customers in the world, and this cannot be done by using the traditional approaches for targeting customers by looking to their regions, age, or gender. However, better approaches to target customers by using online advertising are by analyzing the customers' preference and interest. This can be done by following the principle of “customer-based targeting” that identifies customers' interests by IP address (geographical location), navigation habits, HTTP requests, and user profiles (34).

The authors propose the advertising remote open site agent system (*AdROSA*) for automating banner advertisements. The proposed system does not violate customers' privacy as it does not store customers' details in a database. *AdROSA* analyzes the history of Internet sessions for customers along with the current session. Also, it uses web mining techniques that consist of web content and usage mining. By analyzing the history of customers' sessions and applying clustering techniques, *AdROSA* obtains aggregated sessions (called conceptual spaces) for customers with the same navigation patterns and who click on similar advertisements. *AdROSA* uses HTML content analysis of advertising to recommend the right ad matching with customers' conceptual sessions. For example, if customers belong to sport conceptual spaces, *AdROSA* analyzes advertisements that come under a sport theme and publishes the sport ad banner for customers on their current web page. The authors have tested their system by installing *AdROSA* locally on the polish tourism's website server. The system processes eight sessions and displays advertisements based on users' conceptual space. From the following table, we can see that the user's conceptual space changes based on surfing behavior (page requests). Also, *AdROSA* displays different advertising depending on users' visiting patterns and conceptual spaces.

The click model is a method that shows what the user prefers on web searching or Internet ads. This paper focuses on the previous models' problem, which uses individual queries, including the position and relevance, not a tail query. The authors mention that it is fair to build a new model that uses multiple queries or tail queries, taking in the position, local time, user agent, etc. (35–40).

The paper shows examples of previous models used in web searching and online ads, such as

- *Relevance model*: Relevance probability depends on the fact that the user clicks the URLs on the top; if the user examines the URL, then the click depends on the user's eye tracking if it is relevant.
- *Cascade model*: Depending on that the user examines all the URLs, the click depends on the order or relevance.

The authors suggest in the paper a new model called the general click model (GCM). This model has the following features:

- Multi-bias: Using probability between many attributes.
- Across-query learning: To use one query even if it is new by learning from other queries.
- Extensibility: The ability to add or remove attributes.
- One-pass: Using an online algorithm, the output of one session is entered into the next one.
- Application (ads): GCM outperforms past works.

**Figure 3:**
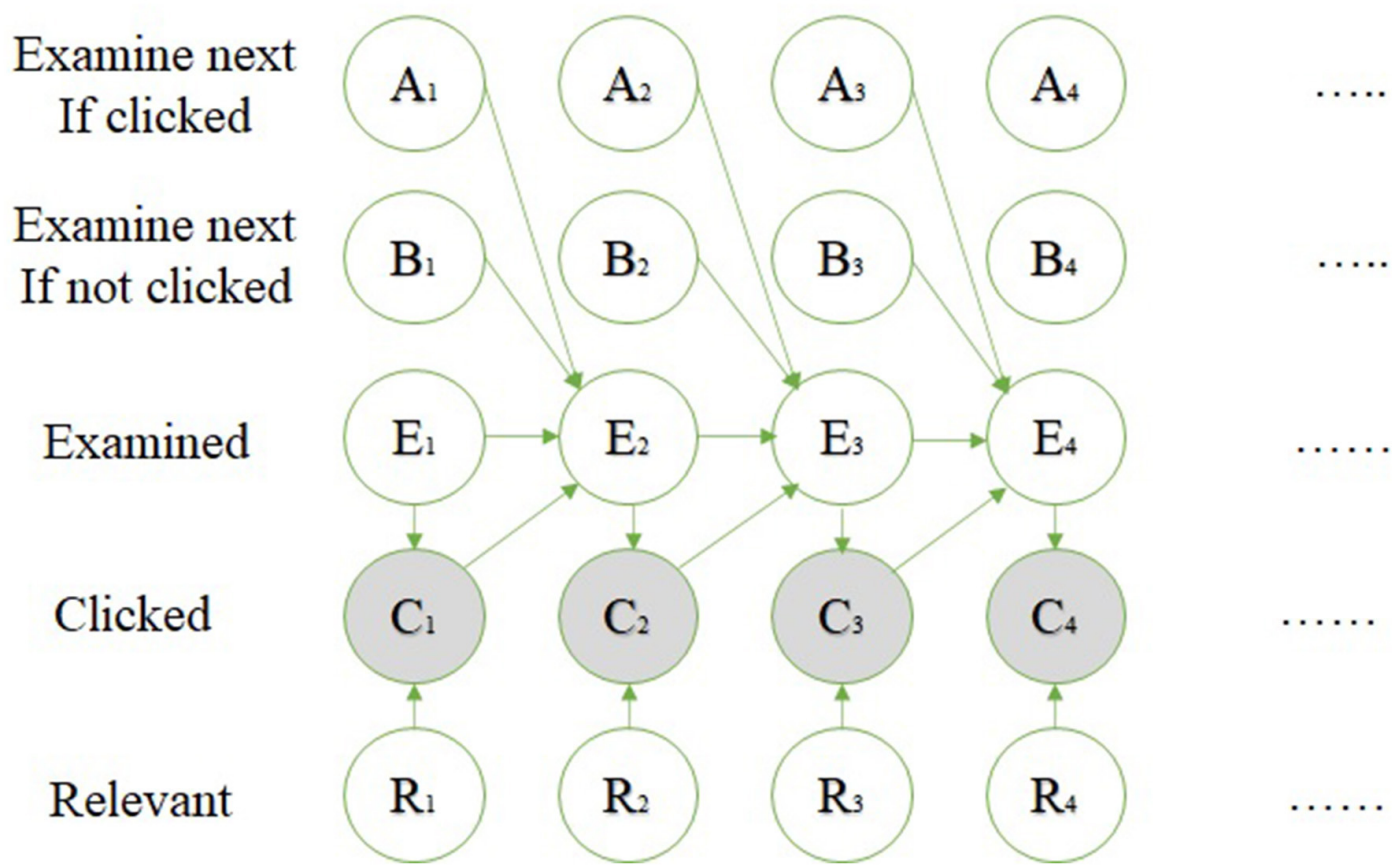
Bayesian network (35).

**Figure 4:**
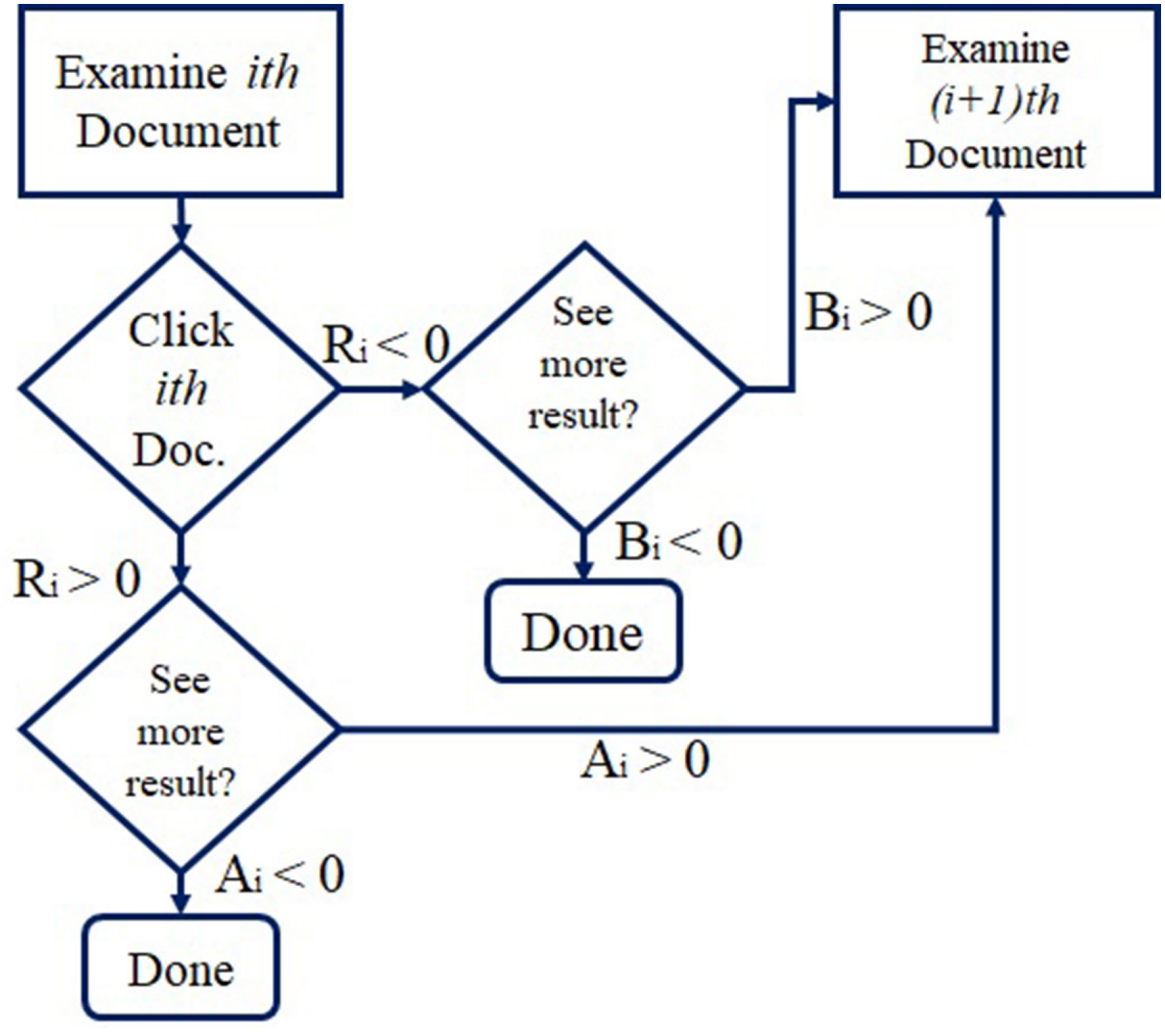
User flow chart (35).

GCM is a general model; the previous models, such as relevance and cascade, are a special case of this model, and it is used in the nested structure, which allows this model to deal with more than the position bias. The structure of this model dealing is shown:

- The outer model: Uses the Bayesian network, shown in Figure 3 and the user flowchart shown in Figure 4.
- The inner model: The query session in this model is categorized in two types:
- User-specific attributes.
- URL-specific attributes.

**Figure 5:**
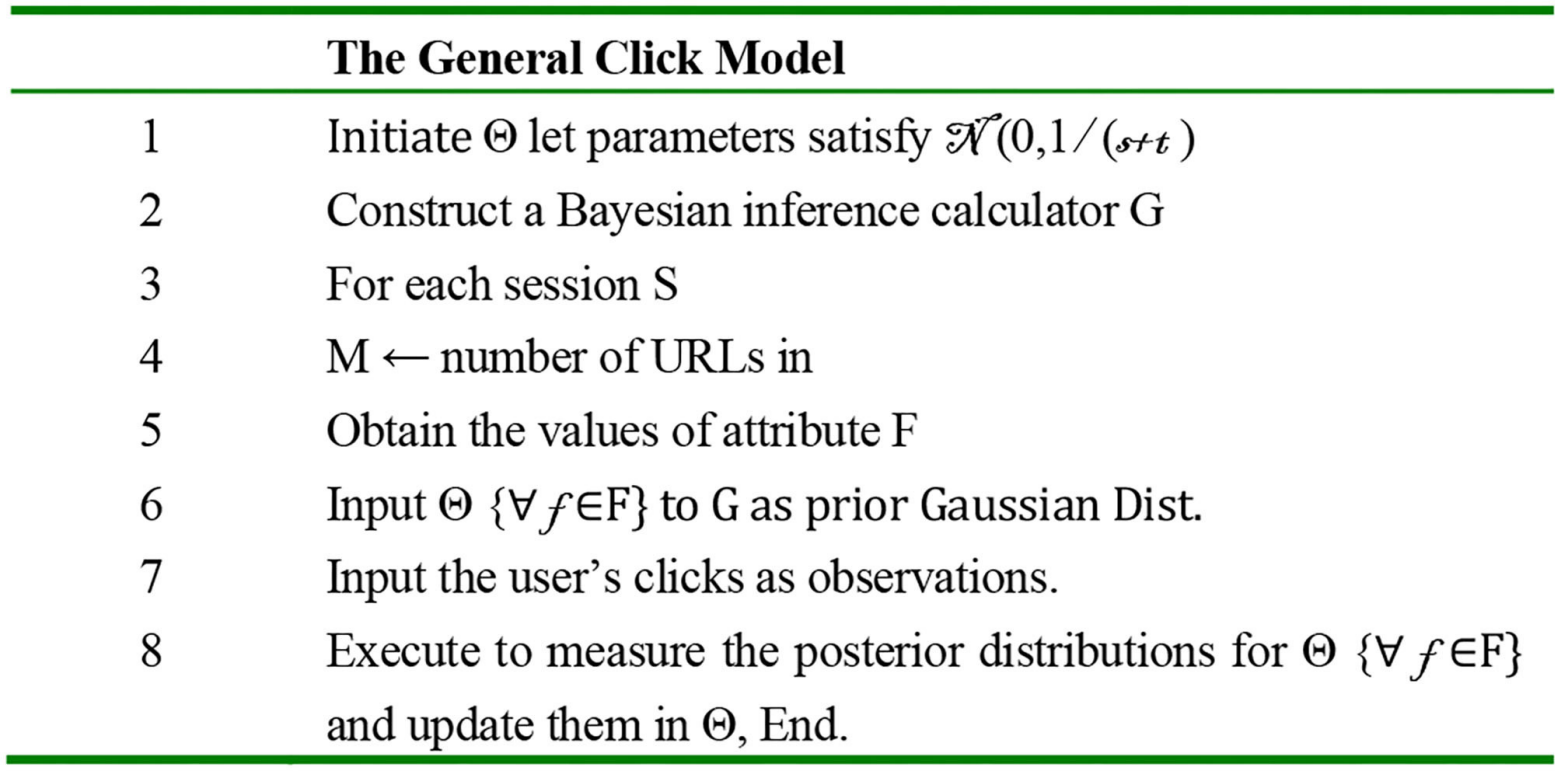
GCM algorithm (35).

The algorithm of online inference using the Bayesian network and the expectation propagation method is clear in Figure 5. The authors performed their experiment using two general data sets: the advertisement data set using 21 attributes and the search data set. The experiments and the results applied on the ads data set are as follows:

- Evaluation on log-likelihood measurements (empirical cross entropy) used to measure one URL impression's accuracy.
- Evaluation on perplexity, which finds the accuracy for the position.
- Evaluation on *R*^2^ by which each 1,000 URLs are blocked.

**Figure 6:**
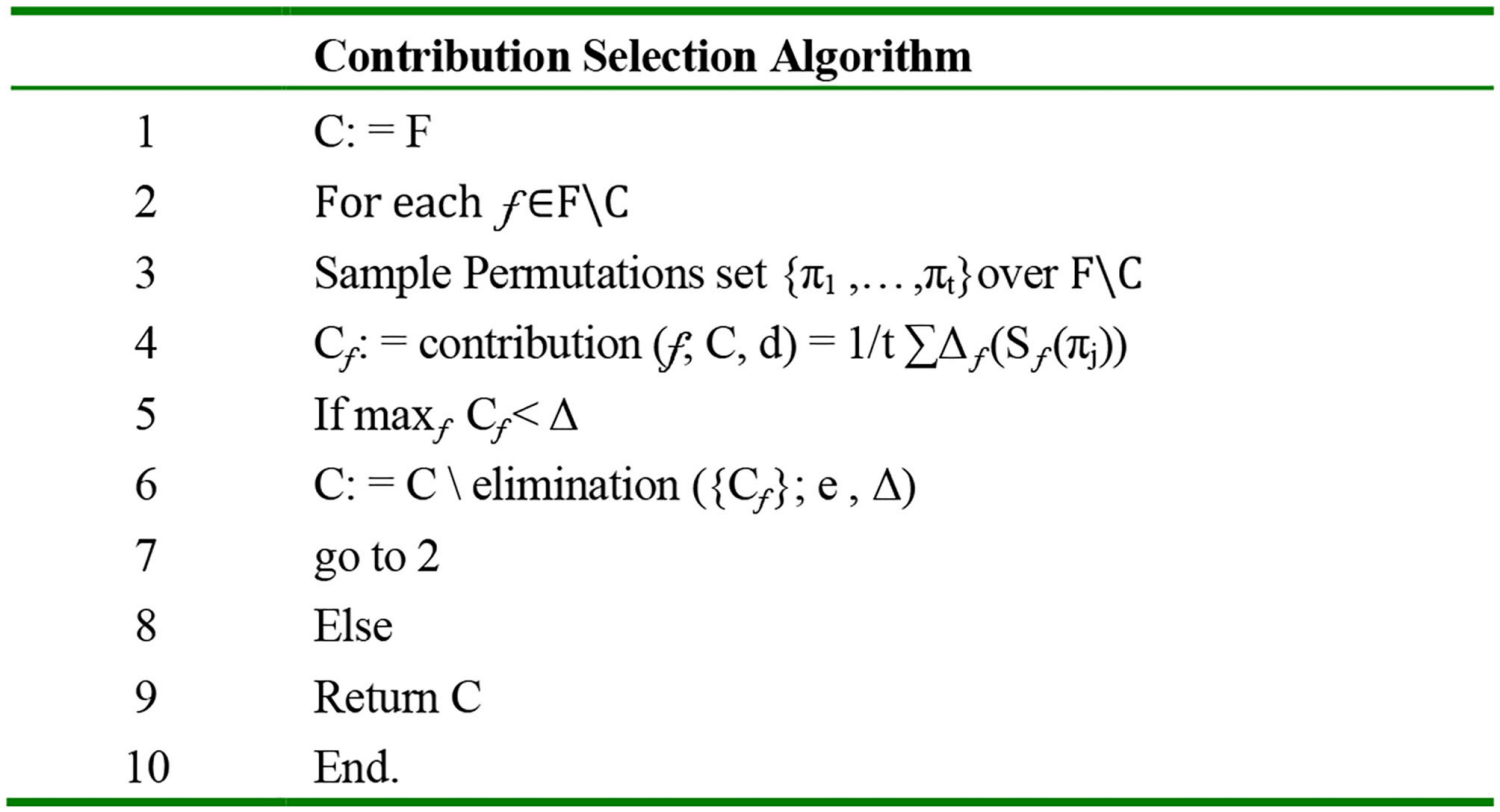
CSA backward elimination algorithm (41).

Another paper represents the contribution selection algorithm (CSA), focusing on the attributes to be selected to maximize classifiers' performance on prior unseen data as one of the feature selections benefits (41). The CSA algorithm use the filter and the wrapper approaches; it is ranked on each step based on Shapley value by a novel ranking method. The Shapley value of the player is a weighted mean over all possible subsets in the margin. The author applies the CSA using either the forward selection or the backward elimination approaches. Figure 6 shows the algorithm. The algorithm was applied to seven data sets, including the internet advertisements data set (ads data set). Eight compared features selection schema used in the experiment are the induction algorithm with no feature selected as the baseline, SVM, different filtering, random forests, forward selection wrapper, and classification with forwarding selection or backward elimination. The results on the ads data set show that the accuracy was between 94 and 96% for all algorithms, but no feature selected by wrapper, the 1NN algorithm, was outstanding.

In another study, random projection (RP) was compared, and it is an attractive approach for dimensional reduction, compared with PCA, a popular approach (42). Five real-world data sets are used in this experiment, including the ads data set. The techniques used in the experiment to compare RP and PCA decision trees (C4.5) are nearest neighbors methods (1NN and 5NN) and linear SVM light. The authors used data sets with small sizes and others with large sizes, and they used low-dimensional data sets and other high-dimensional data sets. The ads data set was high-dimensional and large. They divided the data set into training and test subsets, depending on the size of the data set. The results show that the PCA is expensive computationally but gives more accurate results compared with RP, which is the cheapest computationally but has some good characteristics. Using RP improves performance by increasing the dimensions, doing the best with NN methods, and doing well with SVM. It has a problem with the decision trees (C4.5). In the Table 1, a summary of the research literature's methods and goals is mentioned to overview the methodologies of the literature.

**Table 1:** Literature summary.

**Date**	**Author**	**Methodology**
2016	Alama	Comparison of several Data Mining Algorithms for Internet of Things
2020	Lin	Real-Time Data Mining for Wireless Sensors based on IoT
2018	Yoshiaki Kawase	Queueing System for the process of Bitcoins Chain
2017	Kaur Sahi	Online Experience by Structural Equation Modeling
2016	Peng	Structural Equation Modeling
2015	Neha Derma	Data Mining and Neural Net
2015	Abdallah	Digital Marketing by Structural Equation Modeling
2018	Dijana Oreški	Pre-processing techniques for the evaluation of IoT Intrusion Detection
2020	Balaji	K-Means Feature Location for software artifacts
2017	Jiawei Zhu	Mining Information on Bitcoin Network Data
2015	Kshitij Shah	McAfee Malware detection system IBK, SVM and Adaboost
2020	J. Ni and K. Zhang	SPOON Privacy Protection and task assignment
2020	J. Xiong,	Game theory and Data Encryption
2017	T. Li	Bidding's Privacy by grouping participants
2020	L. Zhu	Collusion Resistance Mechanism in FOG computing
2020	Yang Liu, Tong Feng	Data Aggregation Error Minimization in Privacy-Preserving Crowdsensing

## Research Method

Discriminant analysis is widely used in engineering fields, such as electrical, vibration, and control engineering, but in this study, it is implemented in web usage and IoT (39, 43–45, 47–53). This study's objective lies in selecting a complex advertisement data set from those several hundred attributes and selecting the advertisement from a non-advertisement picture.

**Figure 7:**
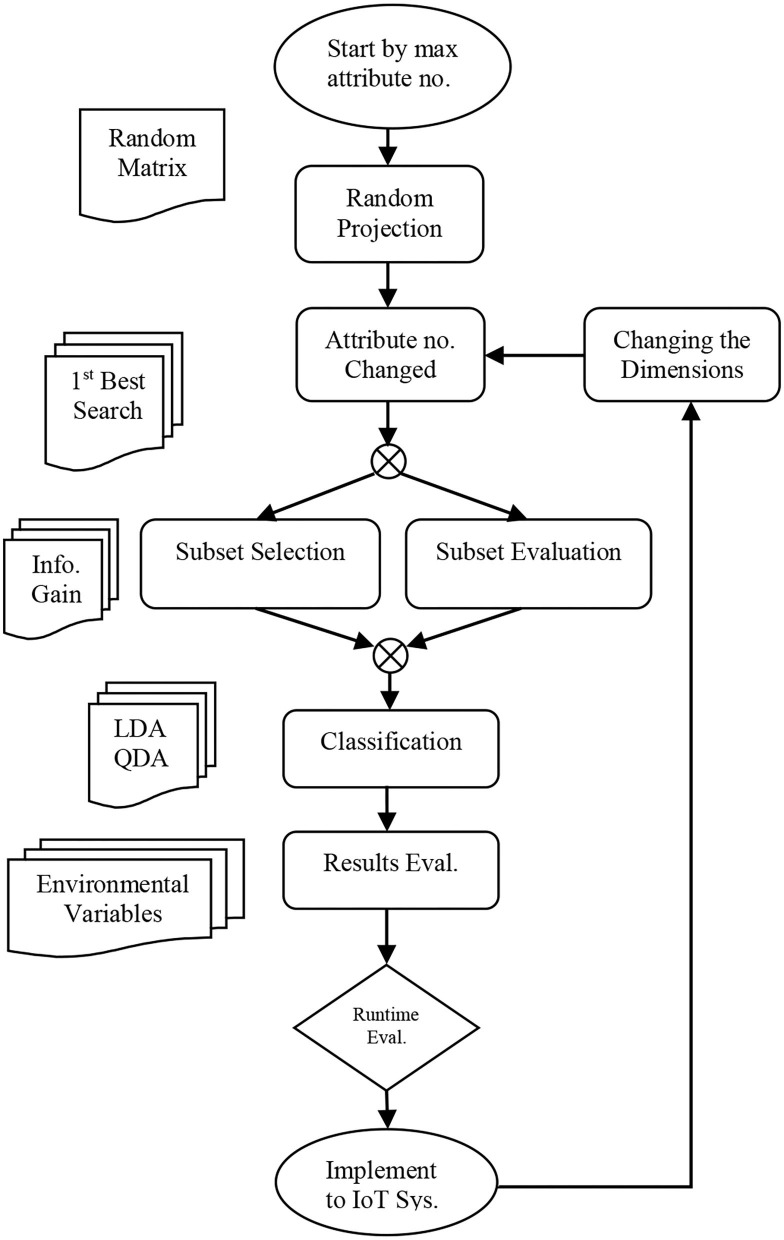
Flow Chart of Research Concept (41).

**Figure 8:**
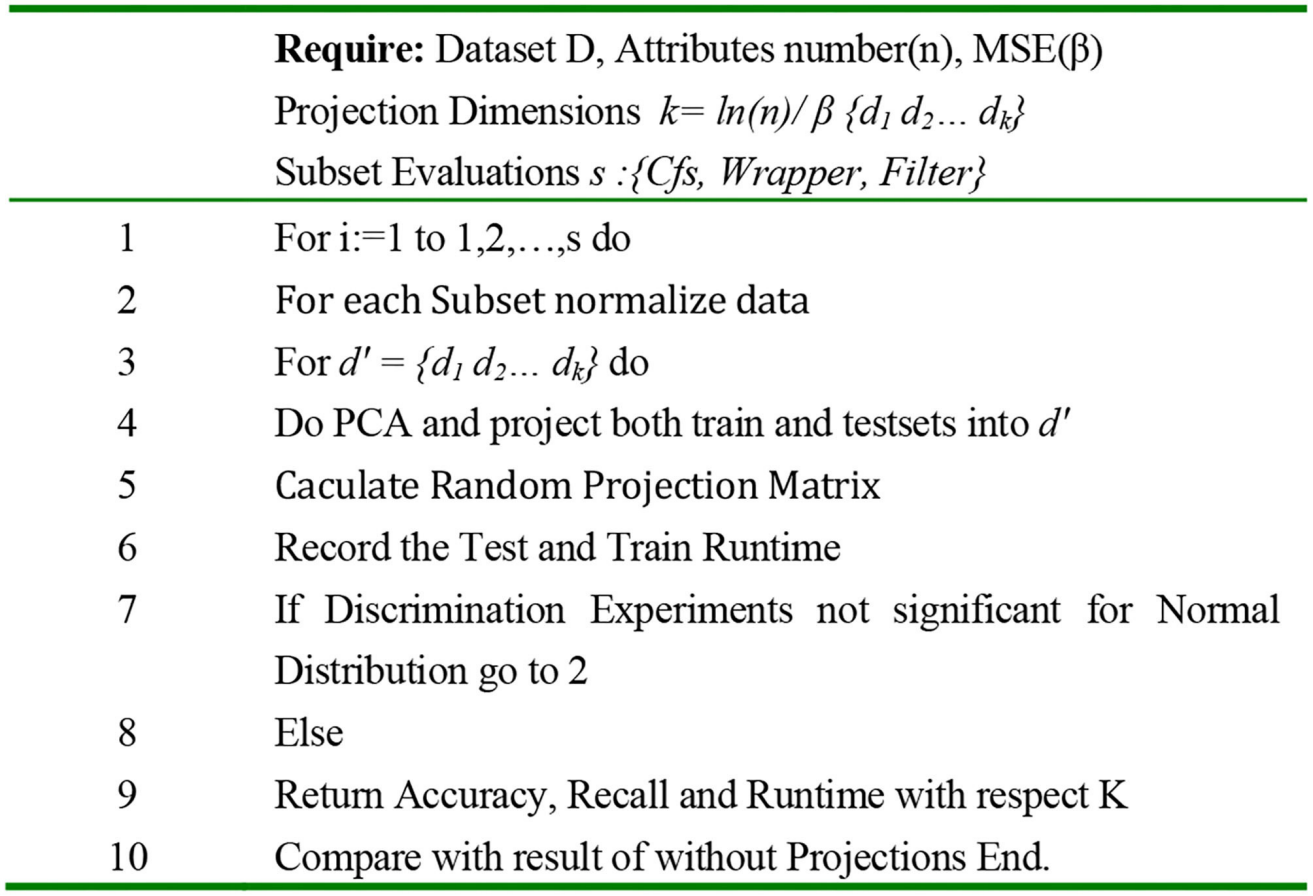
Algorithm pseudocode.

According to Figure 7, the proposed algorithm method is performed in the form of classification problem with subset evaluation and a selection step. It is started by a maximum number of attributes followed by unsupervised filtering with RP, which is supposed to reduce the number of attributes. By reduction of attribute numbers in the Weka, a new subset is sorted with less dimension accompanied with new information gain. By reducing the attributes, it is expected to result in less runtime. The RP (described in Figure 8) filter uses the searching algorithm based on the best first for a lower time budget. Switching the model to new data set with FLDA, the same method as mentioned in the research work of Pang et al., a Gaussian per binary class and concentrate on pooled covariance matrix instead of per multiclass vector-based covariance matrices and means computations, data mining resources are measured and reported (45).

We have additionally tried the quadratic form QLDA for the tuning and sensitivity analysis.

We want to concretely know how the companies that are part of the IoT systems have the possibility of competition to the highest degree for their business? Second, which part of computational resources of their data mining activities are the most important and why: algorithm, runtime, accuracy, data warehouses, services, etc., and finally, is there any model framework for ad selector application available to practically align data mining with the needs of all their stakeholders?

### Data Description

**Figure 9:**
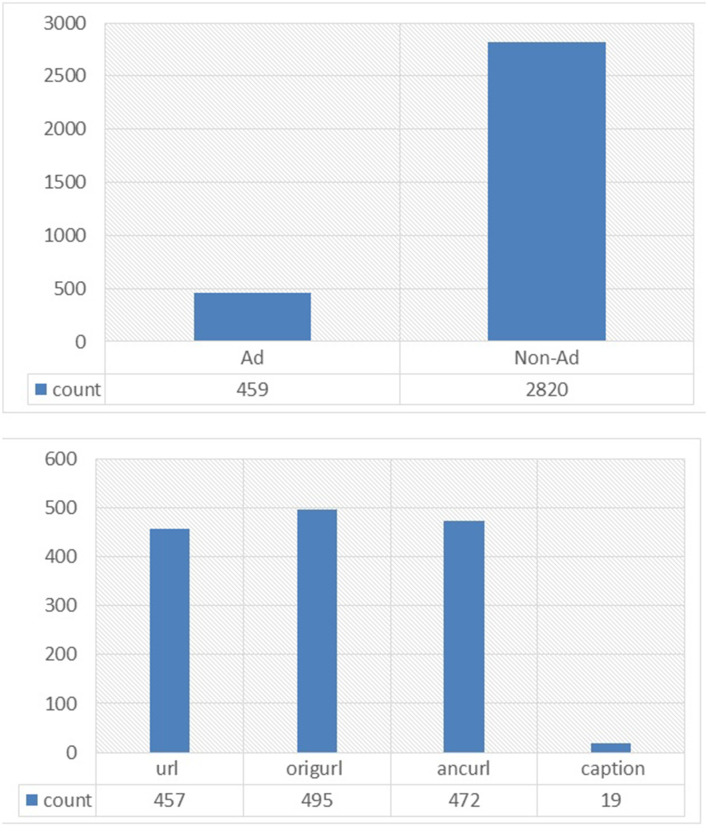
Binomial class distribution.

The Internet advertisements data set (ads data set) collects data used by researchers about advertising in websites, and it can be categorized as a standard, high-dimensional, large, and imbalanced data set. It contains a set of advertisements as images, the geometry of the image, the anchor, the anchor text, and other image properties. The data set available in UCI contained 1,558 attributes and 3,279 instances. The predicted class in this data set was to predict if the image is “ad” or “non-ad.” The ads data set contains 2,821 non-ads and 458 ads as shown in Figure 9.

The ad dataset, as mentioned, contains 1,558 attributes, three of which are continuous. These are height, width, and ratio. There is 28% missing data; some of these data are from continuous attributes, which should be mentioned as “unknown.” The information attributes are classified into four main categories as follows: 457 features are from URL terms, 495 features from origURL terms, 472 features from ancURL terms, and 19 features from caption terms.

### Feature Selection Analysis

**Figure 10:**
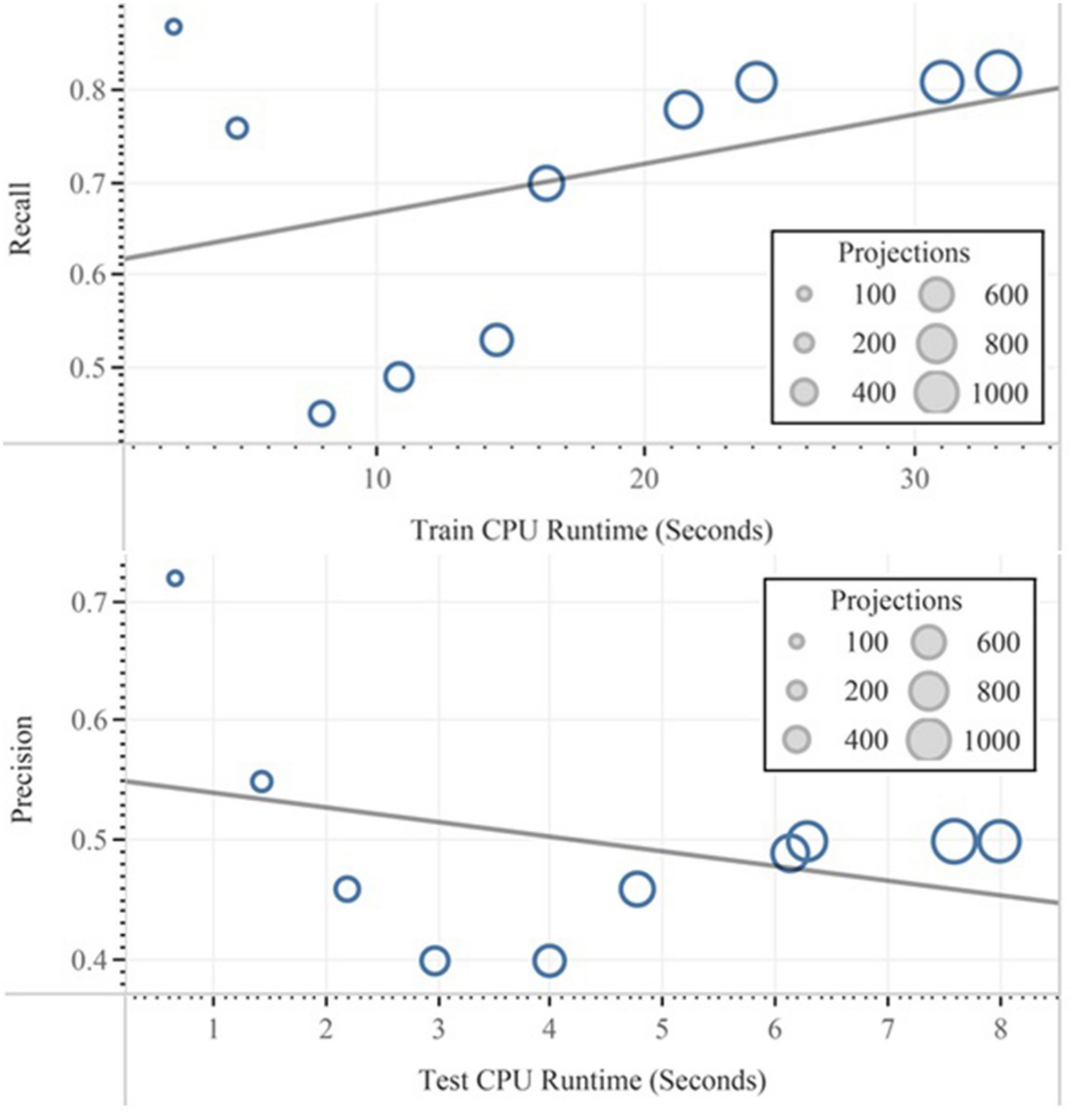
Effect of the projection numbers on precision and runtime implemented with QDA.

In feature selection, techniques are differentiated by their ability to generate other data sets from the original data from which resulted in less mean absolute error rate. In our context, this can be accompanied with the decline of the computational budget based on less runtime as well as reducing of the danger of overfitting for the IoT framework and can be later compared and approved by the above task. The only exception is that our selected data set with 2.7% error and <1% overfitting from the 10-fold cross-validation does not seem like a good candidate for the accuracy enhancement, but it can be efficiently applied for reducing the run time. This might happen on the basis of the anomaly in the distribution of the attribute's categories and also on the basis of numerical attributes. The calculation result in terms of precision and test runtime with the relevant 100 to 1,000 number of projections are represented in Figure 10. The data are filtered, and default settings for the calculation of the QDA were implemented.

The ads data set contains a large amount of data. The data are reduced using the attribute selection method. Feature selection or variable subset selection is the technique of selecting a subset of relevant features for classification. By removing most irrelevant features from the data, feature selection helps improve the performance of classification.

Five techniques of attribute selection were applied to the ads data set. These are Cfs, Info gain, PCA, filter, and wrapper subsets. One of the indicators of the performance of the attribute selection is to determine the number of attributes that were selected by the given attribute selection method. In this manner, the run of the Cfs method results in selecting 24 attributes. The filter subset method gives us the minimum result to only 10 attributes. Unfortunately, the PCA method gives us a big number of attributes reaching 300 attributes. The other two methods (info gain and wrapper) failed to select attributes where it shows a full number of attributes (1,558 attributes). In conclusion, the smaller the number of attributes, the faster the results we get and the easier the model building for classification. This is in the condition of using a variety of data, which is applied in the ads data set.

The results of this work show that the combination of Cfs attribute selection method and the k-nearest neighbor method outperforms any other combination. See the below table (Table 2).

**Table 2:** Classifiers accuracy for the different classification methods of each attribute selection method.

**Attribute selection**	**Classifiers**	**Correctly classified instances**	**Incorrectly classified instances**	**Kappa statistic**
Cfs	Decision tree (J48)	97.23%	2.78%	0.88
	k-Nearest Neighbors (IBk)	**97.76%**	2.24%	**0.91**
	Multilayer Perceptron (NNs)	96.68%	3.32%	0.86
Filter	Decision tree (J48)	97.21%	2.78%	**0.88**
	k-Nearest Neighbors (IBk)	**97.49%**	2.51%	0.90
	Multilayer Perceptron (NNs)	97.39%	2.60%	0.89
Wrapper	Decision tree (J48)	85.11%	14.89%	0
	k-Nearest Neighbors (IBk)	**85.11%**	14.89%	0
	Multilayer Perceptron (NNs)	85.11%	14.89%	0

*Bold values indicates the best True Positive Rate and their kappa statistics*.

### Classification Analysis

Classification is a data mining technique that assigns instances in a data set to target classes. The aim of classification is to accurately predict the target class for each instance in the data. Three different classification methods were used for each one of the three-attribute selections. These are the multilayer perception (NNs), the k-nearest neighbors algorithm (IBk), and the decision tree (J48). The k-nearest neighbors algorithm was set to run with *k* = 1 because there was no significant difference for higher *k* number. The large number of the ads data set allows us to use a Weka default value of 66% training and 34% testing set to apply the decision tree method. This is true for the other two methods of classification adopted in this work. The number of instances used for testing (34%) results in 1,115 instances out of 3,279. We started the analysis of the multilayer perception algorithm based on 1 fold in which the system divides the data automatically into 66% as a training set and 34% as a testing set. For further investigation, we redo the same technique using 10 fold cross validation (Weka default value). Using this technique, we used the whole set of instances, 10% each time as a training set. The results were significant and confirmed that the k-nearest neighbors' algorithm outperforms the other methods. Other indicators for the accuracy of classification techniques are the mean of absolute, the root mean squared, and the relative errors. All of these indicators are calculated using Weka and presented in Table 3. We found that the lowest mean absolute error is found in using the k-nearest neighbors (IBk) method (0.0321). The root mean squared error of it is around (0.14). This method has a root relative square error of (41.89%). An algorithm that has a low error rate is preferred as it has the most powerful classification technique.

**Table 3:** Errors of classifying Ads dataset using different classifiers and attribute selection methods.

**Evaluator**	**Classifier**	**MSE**	**RSME**	**Relative absolute error**	**Root relative squared error**
Cfs	Decision tree (J48)	0.057	0.165	23.22%	46.3%
	k-Nearest Neighbors (IBk)	**0.032**	**0.149**	13.13%	**41.9%**
	Multilayer Perceptron (NNs)	0.039	0.164	16.04%	46.2%
Filter	Decision tree (J48)	0.055	0.163	22.51%	45.95%
	k-Nearest Neighbors (IBk)	0.032	0.147	13.28%	41.2%
	Multilayer Perceptron (NNs)	0.044	0.163	18.08%	45.6%
Wrapper	Decision tree (J48)	0.244	0.356	99.90%	100%
	k-Nearest Neighbors (IBk)	0.244	0.356	99.90%	100%
	Multilayer Perceptron (NNs)	0.244	0.356	100%	100%

Alternatively, the Kappa statistic (46) is used to assess the process for the classification method. It reflects the difference between the actual agreement and the agreement expected by chance; for example, a Kappa of 0.91 means there is 91% better agreement than by chance alone. Using the Kappa statistic criteria, we found that the accuracy of the three classification methods used in this study is substantial because the Kappa statistic for each one is more than 0.88 as shown in Table 3.

## Results and Discussion

As this research concern was partly on the resources relevant to the IoT, it is favorable to represent which part of computational resources of their data mining activities are impacted by the drift concept: algorithm, runtime, accuracy, data warehouses, services, etc., or is there any model framework for ad selector application available to practically align data mining with the needs of all their stakeholders. This is also important to understand the technical difference in crowdsourcing and other data sets.

In Table 4, the experiment results by changing the random projection on the basis of the FLDA are represented. This can be performed by the Weka experimenter module, in which the random projections are set conditionally to get the significant value for the FLDA parameters algorithm. The model performance for QDA as an evaluation method is used to assess model performance for the 10 different RP algorithms used in this work. Table 5 represents the runtime and precision resources for the QDA algorithm. The results on these tables clearly illustrate the strength and weakness of the two methods numerically. These results are imported to the tableau software for the experimentation of these methods in regards to their runtime and accuracy sensitivity by maintaining the mean standard error in a constant Nivea.

**Table 4:** Classifiers accuracy for the FLDA classification method of each attribute selection method.

**Random projections**	**MSE**	**Precision**	**Recall**	**Test runtime**	**Train runtime**
100	0.05	0.92	0.78	0.61	2.48
200	0.06	0.96	0.79	1.08	4.52
300	0.07	0.96	0.82	1.75	7.65
400	0.11	0.95	0.84	2.34	10.74
500	0.16	0.94	0.85	2.87	13.47
600	0.27	0.9	0.84	3.47	16.16
700	0.46	0.86	0.81	3.96	19.54
800	0.46	0.85	0.78	4.33	22
900	0.46	0.85	0.8	5.25	25.86
1,000	0.47	0.87	0.79	5.48	28.67

**Table 5:** Errors of classifying ads dataset using QDA attribute selection method.

**Random projection**	**MSE**	**Precision**	**Recall**	**Test runtime**	**Train runtime**
100	0.07	0.72	0.87	0.65	2.42
200	0.12	0.55	0.76	1.42	4.78
300	0.15	0.46	0.45	2.18	7.92
400	0.17	0.4	0.49	2.96	10.79
500	0.18	0.4	0.53	3.98	14.42
600	0.16	0.46	0.7	4.76	16.27
700	0.14	0.49	0.78	6.12	21.36
800	0.14	0.5	0.81	6.27	24.07
900	0.14	0.5	0.81	7.98	30.97
1,000	0.14	0.5	0.82	7.58	33.06

**Figure 11:**
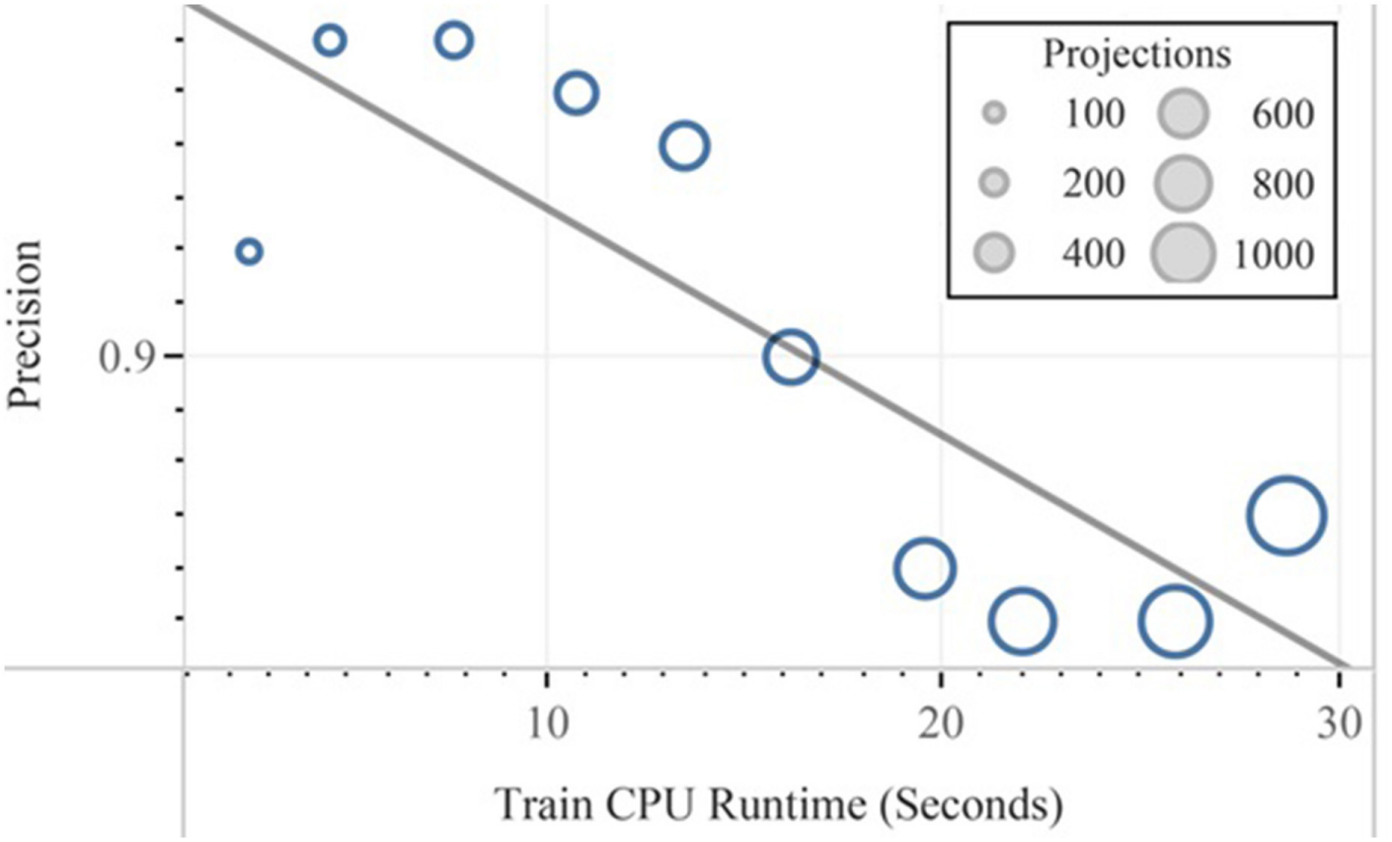
Precision vs. training CPU runtime experimented in QDA.

**Figure 12:**
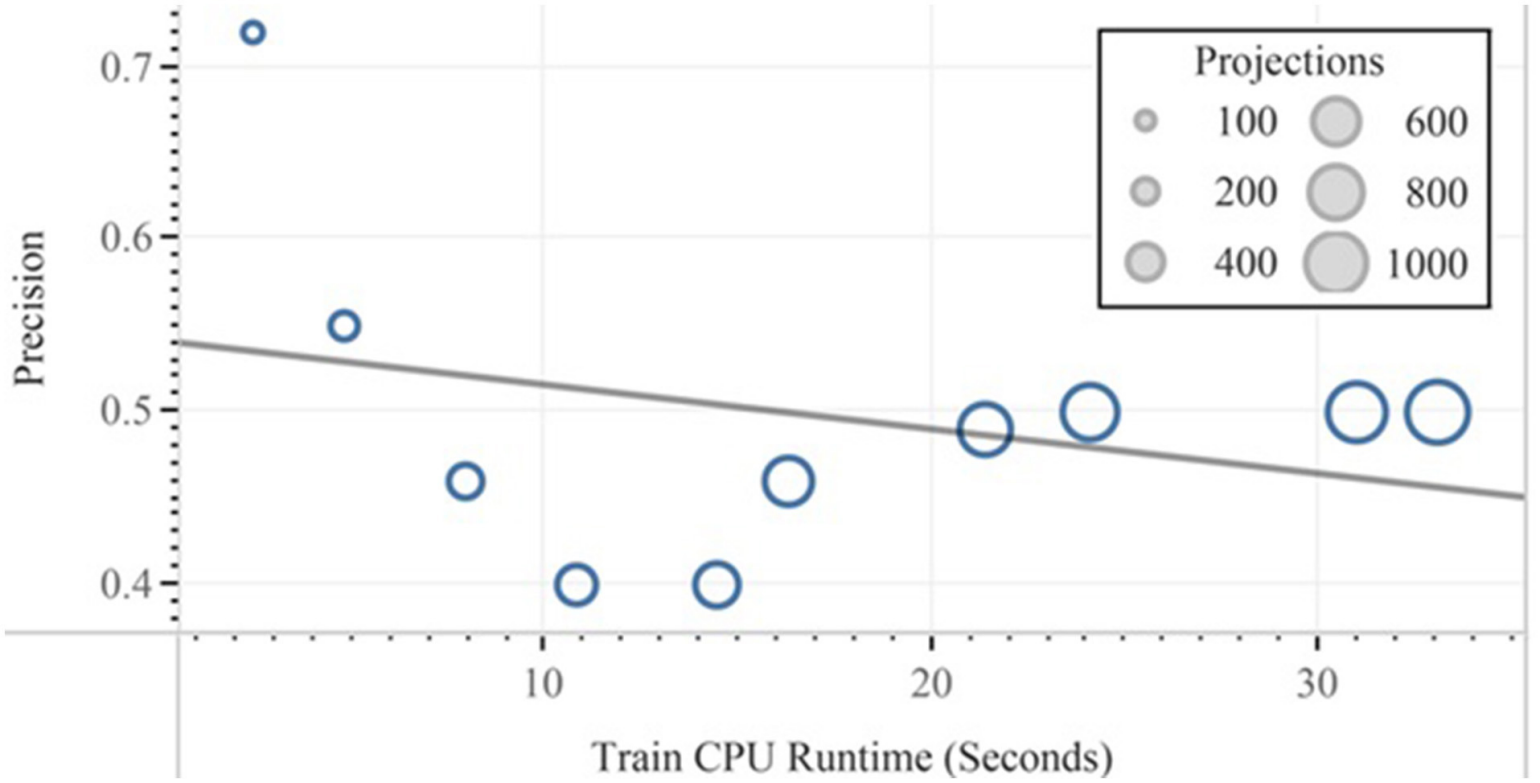
Precision vs. training CPU runtime experimented in FLDA.

Finally, after the feature reduction, subsequent classification with the help of linear and quadratic discrimination analyses for the class “ads” is represented. In Figures 11, 12, the performance vs. runtime of FLDA and QDA are represented where their mean standard errors are equal.

**Figure 13:**
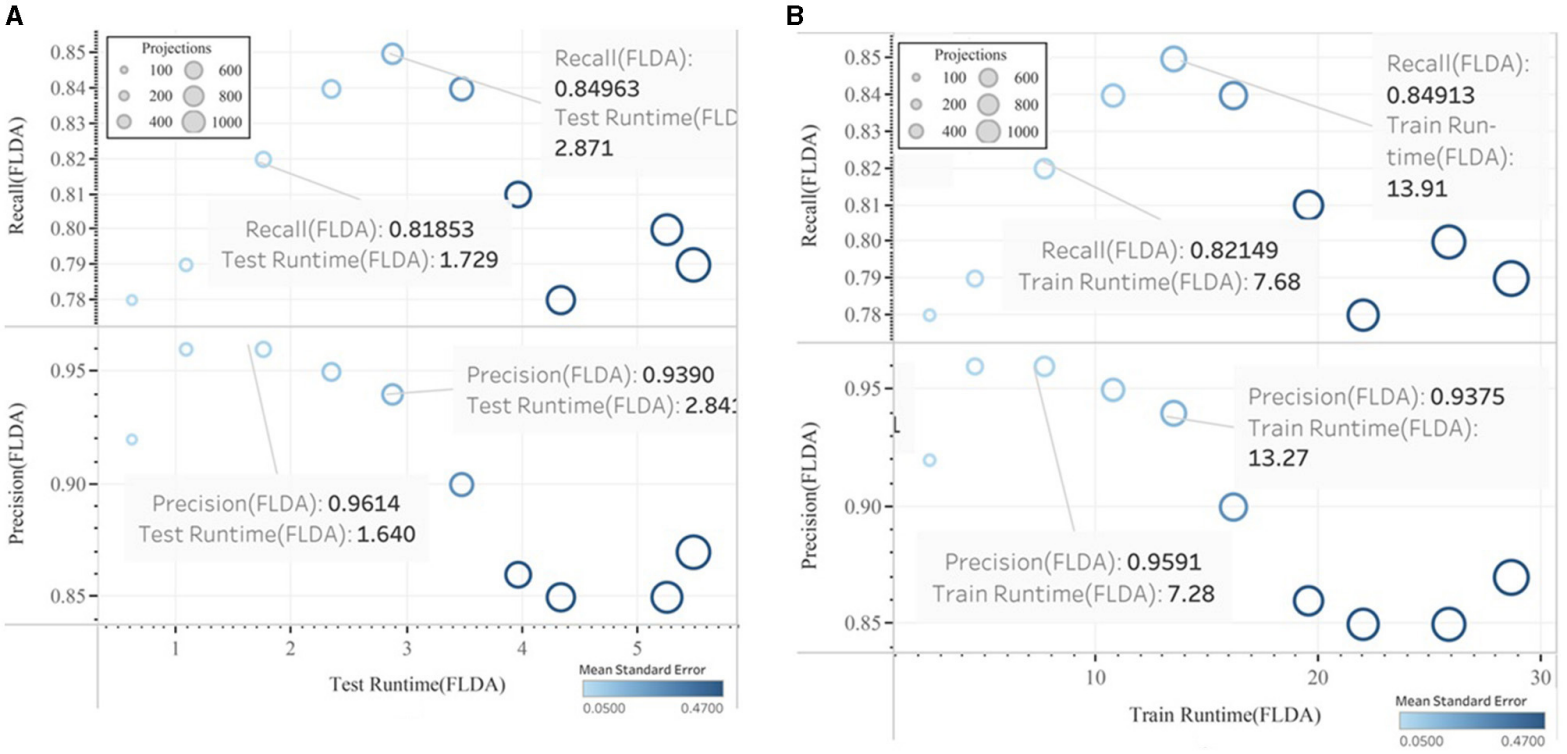
Precision and Recall versus CPU Runtime using FLDA algorithm **(A)** Testing, **(B)** Training.

**Figure 14:**
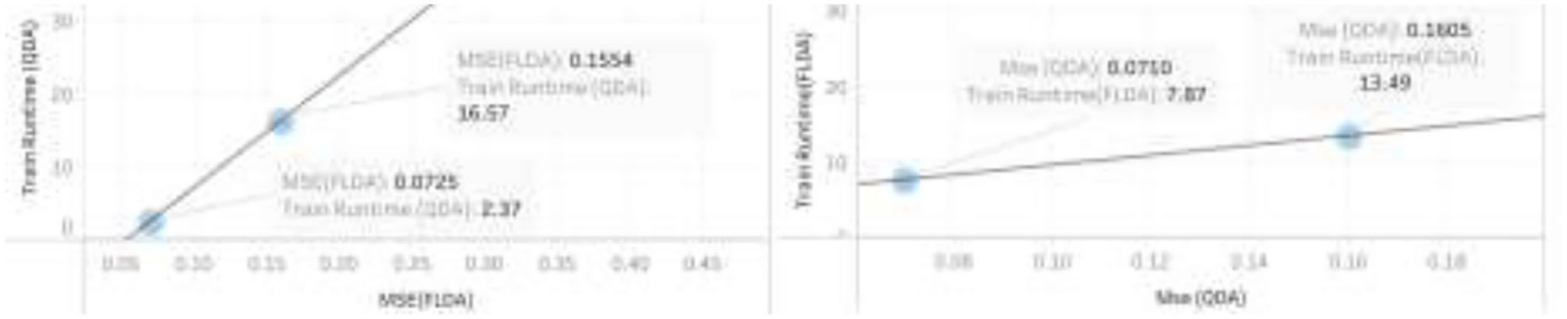
Sensitivity analysis of mean standard error using both QDA and FLDA algorithm.

The results of this work show that, in the combination of FLDA and QDA by equal mean standard error, the FLDA with shorter runtime outperforms any other combination in terms of accuracy, recall, and runtime. The runtime was decreased from 16.57 to 13.49 in addition to the decreasing of the accuracy from 96 to 94% (Figures 13, 14).

## Conclusion

This article studied the typical data mining resources such as precision, runtime and mean standard error for an IoT framework, especially healthcare applications in which reliability, responsiveness, and availability are vital. For this reason, we have applied different classification algorithms based on an Internet advertisement data set. The best algorithm for classifying is found to be FLDA with an accuracy of 94%. It has the mean absolute error at 0.16. The other classification methods gave us satisfactory results but more calculation time; however, in this work, the calculation time was decreased 20 times. These results suggest that, among the data mining techniques tested in this work, FLDA methods can significantly improve the classification methods for use in different areas, especially the IoT. This is important because, with 2% accuracy costs, the runtime can be best achieved. This is very important when there is a reported connection between ads and service reliability or, for instance, trust among empathy and anchor text. This result has been studied sizably by the feature reduction method and results were analyzed by the Fischer linear discriminant analysis in the WEKA Experimenter module. Before recognizing the combination of the two data processing methods, the purpose of the combination should be determined, which can be used to allocate higher process time with low memory for the IoT project and drift learning. If this does not exist, we specify its constituent elements. In collective outsourcing projects, we are not opposed to technological constraints for knowledge orientation. Discriminate analysis provides a progressive framework for assessing classification techniques' performance by changing the RP attributes and other hyperparameters. This cannot only be advantageously coupled with the IoT unless the weka-based experimenter model is implemented. It is vital to include other comparison measures into the evaluation process even though using margin curves for a given data set needed more research work to determine which classification method is the best. What is its optimal decision threshold? It also required using more empirical data sets rather than using only the ads data set. This will be in the benefit of solving real-life problems in healthcare applications.

## Data Availability Statement

The original contributions presented in the study are included in the article/supplementary material, further inquiries can be directed to the corresponding author/s.

## Author Contributions

TG and MH: research concept and methodology. AA, NN, and JA: review and editing. MH and KA: supervision. TG, MH, AO, and KA: original draft. TG, MH, and AO: software and visualization. All authors contributed to the article and approved the submitted version.

## Conflict of Interest

AO was employed by company Tehran Urban and Suburban Railway Co. The remaining authors declare that the research was conducted in the absence of any commercial or financial relationships that could be construed as a potential conflict of interest.

## Publisher's Note

All claims expressed in this article are solely those of the authors and do not necessarily represent those of their affiliated organizations, or those of the publisher, the editors and the reviewers. Any product that may be evaluated in this article, or claim that may be made by its manufacturer, is not guaranteed or endorsed by the publisher.

## Footnotes

^1^Extended Mark-up Language.

^2^Asynchronous Java-scripts.
